# Music and Language in Williams Syndrome: An Integrative and Systematic Mini-Review

**DOI:** 10.3390/bs15050595

**Published:** 2025-04-29

**Authors:** Jérémy Villatte, Agnès Lacroix, Laure Ibernon, Christelle Declercq, Amandine Hippolyte, Guillaume Vivier, Nathalie Marec-Breton

**Affiliations:** 1 Laboratoire LP3C, Université Rennes 2, 35000 Rennes, France; agnes.lacroix@univ-rennes2.fr (A.L.); nathalie.marec-breton@univ-rennes2.fr (N.M.-B.); 2 CeRCA, CNRS-UMR 7295, Université de Tours, 37200 Tours, France; guillaume.vivier@etu.univ-tours.fr; 3 Laboratoire CRP-CPO UR7273, Université Jules Verne Picardie, 80000 Amiens, France; amandine.hippolyte@u-picardie.fr; 4 Laboratoire C2S, Université de Reims Champagne Ardennes, 51097 Reims, France; christelle.declercq@univ-reims.fr

**Keywords:** Williams syndrome, language, music

## Abstract

Individuals with Williams syndrome (WS) are known for their interest in language and music. As producing and comprehending music and language usually involve a set of similar or comparable cognitive abilities, the music–language relationship might be of interest to better understand WS. We identified, analyzed, and synthesized research articles on music and language among individuals with WS. Three different databases were searched (SCOPUS, PubMed, PsycInfo). Eight research articles were identified after screening, based on title, abstract and full text. In this integrative–systematic review, we assess methodologies, report findings and examine the current understanding of several subdimensions of the relationship between music and language. The findings suggest that basic musical abilities such as tone, rhythm and pitch discrimination are correlated with several verbal skills, particularly the understanding of prosody. Musical practice seems to benefit individuals with WS, in particular for prosody understanding and verbal memory. A correlation was also observed between emotional responsiveness to music and verbal ability. Further studies are needed to better characterize the relationship between music and language in WS. The clinical use of musical practice could be of interest in improving prosodic skills and verbal memory, which deserves extended experimental investigation.

## 1. Introduction

Williams syndrome (WS), also known as Williams–Beuren syndrome, is a rare neurodevelopmental disorder with a prevalence between 1 in 7500 and 1 in 10,000 (Strømme et al., 2002). Since the first case reports in the 1960s, WS has been described as a very specific mix of physiological and cognitive impairments (Berdon et al., 2011; Beuren et al., 1962; J. C. P. Williams et al., 1961). In the 1990s, the genetic origin of the disorder was demonstrated, with WS being the result of the hemizygous deletion of 26–28 genes on chromosome 7q11.23 (Ewart et al., 1993; Morris et al., 1988; Strømme et al., 2002). Individuals with WS usually display a very specific pattern of personality, intellectual disability, and cognitive development. They are sometimes described as highly social or overfriendly and empathetic, despite a tendency to generalized anxiety and specific phobias. Their behavior, emotional reactions, and specific abilities in relation to some stimuli (e.g., music) might also be characteristic. Their general IQ level varies between mild and moderate intellectual disability, with notable difficulties when it comes to visuospatial tasks (Farran & Jarrold, 2003; Mervis et al., 2000). Intellectual impairment in WS is not uniform. Researchers and practitioners have long emphasized that verbal cognition is a domain of relative strength, despite not being spared from impairment. Still, there is an apparent discrepancy between general, visuospatial, and verbal abilities in individuals with WS, and it is one of the key features of their cognitive functioning.

In addition, another widely reported specificity is a marked attraction and hypothesized specific ability for music (for a review see Thakur et al., 2018). Music can be defined as “the art of combining sounds with a view to beauty of form and expression of emotions” (Concise Oxford Dictionary) and, similar to language, “is a human universal involving perceptually discrete elements organized into hierarchically structured sequences” (Patel, 2003). Numerous cognitive abilities are necessary to perceive and produce music, some of which have been particularly studied in WS. For example, a number of studies were dedicated to the processing of pitch (i.e., perceptual correlate of the periodicity of an acoustic waveform, the attribute of auditory sensation in terms of which sounds may be ordered on a scale extending from high to low, Moore, 1995; Oxenham, 2012), tone (i.e., the quality of a sound, as determined by pitch, quality, and strength.) and rhythm (i.e., cadence or long-term similar structure of similar sounds). Closely related, the concept of musicality corresponds to attitudes toward music and includes several subdimensions. In relation to WS, Thakur et al. (2018) proposed four subdimensions of musicality: affinity for music, experience with musical practice (e.g., involvement in band, choir), musical engagement (i.e., time spent playing or listening to music) and artistry (e.g., creativity, expressivity, sensitivity, emotionality).

The observation of cognitive particularities for both music and language in WS is particularly intriguing. Cognitive sciences have a long-standing interest in music and how it relies, more or less, on similar cognitive abilities to language (Arbib, 2013; Slevc, 2012). Considering that music and language are both communication systems, involve the processing of auditory stimuli and a somewhat comparable ability to process syntax, early thinkers like philosopher Jean-Jacques Rousseau or biologist Charles Darwin had already discussed their similarities. Modern theorists have emphasized their common evolutionary origins (Besson & Schön, 2001; Brown, 2001; Jackendoff, 2009). Arguably, some of the features of music and language are identical and involve similar cognitive abilities, while other features are comparable, although relying on relatively distinct cognitive abilities, and some are clearly distinct (Brown, 2001; Jackendoff, 2009; Lerdahl & Jackendoff, 1996; Mcmullen & Saffran, 2004; Pinker, 1997). Distinguishing between similar, comparable and distinct aspects of music and language is not an easy task. However, a broad classification can be attempted based on current knowledge. Probable common aspects are vocalization, affective prosody, auditory temporal processing, tone and pitch discrimination (Brown, 2001; Deutsch et al., 2004; Kraus & Slater, 2015; Pfordresher & Brown, 2009; Zheng & Samuel, 2018). For example, auditory temporal processing is necessary for rhythm discrimination, which is itself a crucial feature of both music and language (Fiveash et al., 2021; Kraus & Slater, 2015). Comparable aspects between music and language may include the use of a discrete set of units, combinatoriality, phrasing and the involvement of an auditory memory system (Brown, 2001; Fennell et al., 2021). Finally, distinct aspects would include the conceptual meaning of verbal lexicon, which has no equivalent in music, and large differences in syntactic organization and purpose (Brown, 2001; Jackendoff, 2009; Patel, 2003). In the general population, numerous works show that language and music processing involve partly overlapping brain areas and cognitive structures (Fadiga et al., 2009; Koelsch et al., 2004; Mampe et al., 2009). Musical practice seems beneficial for various aspects of cognitive development, particularly in relation to language (R. L. Gordon et al., 2015; Hallam et al., 2011; Linnavalli et al., 2018; Neves et al., 2022; Ong et al., 2024; Protzko, 2017; Sadiqzade, 2024; Schellenberg & Weiss, 2013; Swaminathan & Schellenberg, 2020). Finally, in relation to neurodevelopmental conditions, music is often considered as a potent clinical tool, with various cognitive benefits (Gold et al., 2004; Mastnak & Neuwirthová, 2017; Reis et al., 2003; Shi et al., 2016; Whipple, 2004).

With regards to WS, it is possible that music and language are characterized by both comparable and/or intriguing patterns of strengths and weaknesses (Holinger et al., 2005; Levitin et al., 2004). As already mentioned, language, although good compared to overall intellectual level, is still far from being fully preserved. Overall, productive phonological ability, phonological short-term memory and verbal fluency are strengths (Jarrold et al., 1999; Klein & Mervis, 1999; Miezah et al., 2020; Vicari et al., 2004; Wang & Bellugi, 1994). Individuals with WS are fluent, talkative and enjoy discussion, features perhaps related to their prosocial personality. Classically, their lexical knowledge has been regarded as good; however, current evidence suggests a more complex depiction. Children with WS produce more words than one could predict based on their mental age (MA), which raises the question of whether their production ability would outperform their comprehension ability (Singer Harris et al., 1997). They perform well in tasks involving picture–spoken word association, but when asked to produce the name of a depicted object or action they are not better than MA-matched counterparts (Bello et al., 2004). Receptive vocabulary, the set of words they can understand, is better than productive vocabulary, the set of words they use regularly (Siegmüller, 2004; Thomas et al., 2006; Vicari et al., 2004; Volterra et al., 1996). Still, even receptive vocabulary can be impaired when semantic similarity between targets and distractors increases (Nazzi et al., 2005). A recent meta-analysis (Romero-Rivas et al., 2023) concludes that individuals with WS have worse lexical–semantic skills than individuals with typical development (TD), despite outperforming individuals with other cognitive disabilities.

Seemingly, individuals with WS may be better characterized by a strong interest in language rather than by actual language capability. Interestingly, their musicality (i.e., attitude toward music) could be comparable to a strong enjoyment for music and an interest in practice but not particularly good formal musical skills (Thakur et al., 2018). This strong interest versus impaired ability in both language and music deserve further exploration. On another note, prosody, namely stress, intonation, duration, and intensity that pertain to a sequence of phonemes, is often considered impaired. Early development of prosodic function is delayed in children with WS, and by adulthood, they have difficulties segmenting perceived sentences according to prosody (Loveall et al., 2021; Nazzi et al., 2003; Stojanovik, 2010). Their speech production may also be characterized by atypical prosodic patterns (Ito & Martens, 2017). Considering that prosody is sometimes referred as “the music of speech”, its relationship with musical cognition in WS might also be of interest. For example, there is a question of whether musical affinity, or musical engagement (i.e., time spent listening or playing music, Thakur et al., 2018) mediate the extent of prosodic impairment. Finally, music carries a strong emotional value. WS is also characterized by impaired emotional processing, with delayed recognition of vocal emotion and bias toward positive emotional cues over negative one (Järvinen-Pasley et al., 2010; Plesa-Skwerer et al., 2006). Music might be helpful to better understand how individuals with WS process auditory stimuli that carry an emotional valence.

A number of articles have been dedicated to music and its relationship with language in WS, but to date, no formal review of the topic has been conducted. Only Thakur et al. (2018) have published a review about musicality, but without a specific focus on language. We aimed to fill this gap by investigating the relevant literature. We also suggest directions for future research, based on the current knowledge.

## 2. Materials and Methods

The present paper aims to explore the relationship between language and music in WS by drawing on a combined methodological approach that integrates elements of both systematic and integrative reviews. Systematic review can be defined as a “review of a clearly formulated question that uses systematic and explicit methods to identify, select, and critically appraise relevant research, and to collect and analyze data from the studies that are included in the review” (Moher et al., 2010). One of the objectives is to avoid certain biases of traditional literature reviews, in particular when selecting the articles to consider. A systematic review usually includes scientific reports that followed similar methodologies or report similar outcomes. On the other hand, an integrative review covers a wider range of research. Articles included may have only a fairly general theme in common and use very different sets of methodologies. The value of integrative reviews stem from the greater perspective they give on a phenomenon. They are useful for highlighting relationships between works devoted to a given theme, but that are too different methodologically or theoretically to be included in a systematic review.

This review borrowed from the aims and methods of both integrative and systematic reviews. Considering that the link between language and music in WS is a rather broad topic, approached through a wide variety of methodologies and research objectives, an entirely systematic review was not feasible. Therefore, the integrative review approach was necessary to capture the diversity of the existing literature. Nonetheless, to prevent biases and enhance its quality, we also included some of the principles of systematic reviews. Specifically, we followed the Preferred Reporting Items for Systematic Reviews and Meta-Analysis guidelines (PRISMA, Moher et al., 2015), which define a number of important points to consider when carrying out a systematic review.

### 2.1. Search Strategy

We conducted an electronic search from the databases SCOPUS, Pubmed and PsycInfo using the terms “Williams syndrome”, “music”, “language” and “verbal”(see Supplementary Material at (https://osf.io/prc98/, accessed on 24 April 2025). The eligibility criteria were set as follows:

- Date range: published before 1 February 2023
- Subject: Williams syndrome
- Subject: Music
- Subject: Language or verbal
- Published in a peer-reviewed journal
- Published in English
- Not a review article (as required by PRISMA)

### 2.2. Data Analysis

**Figure 1:**
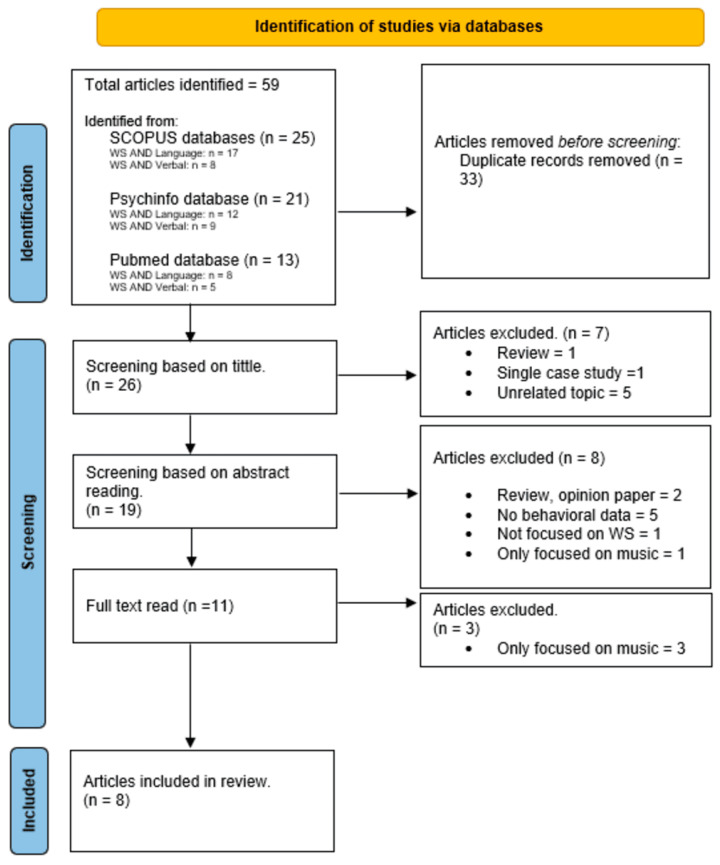
PRISMA flow diagram including the number of studies identified, screened, assessed for eligibility and included in the final review.

Search results were managed with Zotero (https://www.zotero.org/). Once all databases were searched and duplicates removed, a first step was to screen the articles based on the title and abstract. A second screening was then performed based on full-text reading. The full list of exclusions based on both first and second screening steps is provided in Figure 1. Two of the authors carried out the screening in parallel to avoid omissions and errors and they agreed on the final list of articles selected. Thereafter, the co-authors independently extracted data using a data extraction form (see Supplementary Materials at https://osf.io/prc98/, accessed on 24 April 2025) freely inspired by the work of Thakur et al. (2018). Five types of information were extracted: methods, participants, tasks and outcomes, results, discussion and implications. Once completed, the data extraction form provides a synthesis of the corresponding article, depicting its main topics, objectives, results, potential biases and limitations. It also highlights the aspect of musical cognition and musicality that the article focused on. Based on Thakur et al.’s (2018) recommendation, we investigated four subdimensions of musicality: (1) affinity for music, (2) experience with musical practice (e.g., involvement in band, choir), (3) musical engagement (i.e., time spent playing/listening to music) and (4) artistry (e.g., creativity, expressivity, sensitivity, emotionality). In addition, because these sub-dimensions did not accurately describe the objectives of some of the selected studies, we added a fifth sub-dimension called *music perception*.

## 3. Results

A total of eight articles met the criteria for inclusion. These articles were classified according to their main topic of interest. Four different topics of interest were identified: (1) tonal, rhythmic skills and overall language ability (one article), (2) pitch discrimination and prosody (three articles), (3) musicality and verbal memory (two articles) and musicality and emotional processing (two articles) (see Table 1). 

### 3.1. Synthetized Findings

#### 3.1.1. Tonal, Rhythmic Skills and Overall Language Ability

The work of Don and collaborators (1999) was the first article with a specific focus on language and music perception in WS. Their main aim was to assess whether basic musical abilities were correlated to an array of verbal skills in children with WS and without a history of formal musical practice. A specific subsection is dedicated to this work, as its objectives and methods differ slightly from those of other studies. Basic musical skills were assessed using the tonal and rhythm subtests of the Primary Measures of Music Audiation (PPMA, E. E. Gordon, 1979). As expected, children with WS performed similarly to MA-matched TD children, although their performance was lower compared to children with TD matched for chronological age (CA) (for comparable results see Hopyan et al., 2001; Lense & Dykens, 2016). Both children with WS and MA-matched children with TD performed better on the tonal subtest than on the rhythm subtest of the PPMA, supporting the hypothesis of a general preservation of basic musical abilities. Children with WS also did better on musical tests than predicted based on their full scale, verbal, or performance IQ. Verbal evaluation included a range of verbal tests of various difficulty levels. Don et al. (1999) assessed auditory closure, verbal fluency, auditory attention and working memory. Their hypothesis of a relationship between verbal and musical perception skills is supported by moderate correlations between most verbal and musical tests. This correlation was found for both children with WS and TD. Verbal fluency was the only ability not correlated to tonal and rhythmic skills. Don et al. (1999) provided an overall promising exploratory study. It has subsequently been proposed that impaired rhythmic ability compared to CA matched children with TD is a common feature of several neurodevelopmental disorders, and that in Williams syndrome it may be linked to their atypical auditory attention profile (Kasdan et al., 2022; Lense et al., 2021). Unfortunately, to date, the influence of tonal and rhythmic skills on verbal cognition among children with WS has not been investigated further.

#### 3.1.2. Pitch Discrimination and Prosodic Skills

In daily life, prosody conveys a substantial amount of meaning. For example, intonation contrast is necessary to distinguish questioning and declarative sentences in many languages (e.g., English, French, Spanish), and rising intonation may indicate a question while falling intonation expresses a statement. In individuals with TD, some findings indicate that musical ability and practice improve prosodic skills (Besson et al., 2011; Magne et al., 2006). Regarding WS, three articles addressed the relationship between pitch discrimination and prosodic skills (Martínez-Castilla et al., 2019; Martínez-Castilla & Sotillo, 2014; Kitamura et al., 2020). None were interested in similar aspects of music. Two (Martínez-Castilla & Sotillo, 2014; Kitamura et al., 2020) were dedicated to music perception and investigated the ability to process pitch and understand it as a distinct perceptive element. The last one (Martínez-Castilla et al., 2019) was interested in experience with musical practice and musical engagement. Overall, they provide evidence suggesting that, in WS, pitch and prosody are processed through overlapping mechanisms. The work of Martínez-Castilla et al. (2019) also suggest that musical practice may benefit individuals with WS by enhancing prosodic processing, particularly in the comprehension of intonation.

The works of Kitamura et al. (2020) and Martínez-Castilla and Sotillo (2014) addressed pitch discrimination ability and its relation to language.

On the one hand, Kitamura and colleagues were mainly interested by the specificity of language functioning in WS and how the use of verbal response in pitch discrimination tasks might cause bias. They hypothesized that studies using verbal answers may underestimate the pitch processing ability of children with WS. They designed a pitch discrimination task in which participants had to indicate whether the second of two piano tones was higher or lower than the first one by moving a doll on a small staircase. In line with their hypothesis, children with WS performed significantly better when using this nonverbal response device than a verbal one. In addition, they argue that, contrary to children with TD, pitch discrimination ability in children with WS may not develop in parallel to language skills, or at least atypically. If pitch discrimination and verbal ability develop in parallel, one would expect better pitch discrimination in children with the highest verbal mental age (VMA). Yet, they observed similar pitch discrimination ability for children with WS, no matter their VMA. Although this is an intriguing result, the small sample used in this study (i.e., 11 children with WS) calls for caution. That being said, Kitamura et al. (2020) developed an innovative nonverbal pitch discrimination task, and their results suggest that atypical development of pitch processing and language ability in WS deserves further investigation.

On the other hand, Martínez-Castilla and Sotillo (2014) aimed to assess whether pitch processing and prosodic ability are related to each other. They used verbal and pitch discrimination tasks to determine whether they correlated in both individuals with WS and CA-matched individuals with TD. The participants heard pairs of items, either pitches or verbal material, and had to orally indicate whether the items were similar or different. Two different types of verbal items were used, isolated words and short sentences. Both items originated from the Spanish version of the Prosody in Speech communication (PEPS-C) battery (Martínez-Castilla & Peppé, 2008). The main results revealed a significant correlation between pitch and prosody discrimination for isolated words in individuals with WS. Nonetheless, this correlation was significant only when the verbal task involved prosody discrimination for isolated words (e.g., the word “cake” spoken with a rising or falling intonation) and not when it used short sentences. Martínez-Castilla and Sotillo (2014) argue that correct understanding of prosody for isolated words only involves pitch processing, whereas for short sentences it also involves other parameters such as loudness and length. For example, understanding whether the isolated word “cake” was intended as a question or affirmation only involved processing of intonation that was rising in the first case and falling in the second. However, in a short sentence, contrast in length and loudness also need to be correctly segmented to extract the meaning. This can be demonstrated in the following example: “pink and black and green socks”. To understand whether the two sock pairs are either a first pink and black and a second green, or a first pink and a second black and green, intonation alone is not sufficient. Length and loudness contrasts are necessary, both in the English and Spanish languages (i.e., the original language of the study). Therefore, these results emphasize a relationship between pitch processing and intonation, but not with other prosodic parameters.

Regarding the influence of musical practice, the study of Martínez-Castilla and collaborators (Martínez-Castilla et al., 2019) provides encouraging results. The authors compared musically trained and untrained participants with WS and TD. They used several items of the PEPS-C battery and observed that musical practice correlates with several subdimensions of prosodic ability. Extending the results of Martínez-Castilla and Sotillo (2014) they reported that musically trained individuals with WS are better than their untrained counterparts at tasks involving intonation processing (e.g., discriminating between rising and falling intonation). Such an effect was independent of VMA. Typically, musically trained individuals with WS performed better at understanding whether single words were pronounced in a declarative or affirmative manner. They were also better at understanding short sentences when intonation had decisive significance. The results were less conclusive when discriminating short sentences based on mostly duration, lengthening and pause of the prosody. In particular, musical practice might not be sufficient when it comes to segmenting sentences and understanding word boundaries. Considering that segmentation and boundaries are the greater difficulties of individuals with WS, it is not surprising that they are harder to address (Loveall et al., 2021; Nazzi et al., 2003). The authors also suggest that musical practice might not be helpful in individuals with stronger prosodic impairments. Nonetheless, their results clearly suggest that musical practice might have some clinical value and call for further investigations.

#### 3.1.3. Musicality and Verbal Memory

In TD, it is often accepted that memory for verbal information and sounds share processing resources (Berz, 1995; V. J. Williamson et al., 2010). Music improves information transfer from working memory to long-term memory (McElhinney & Annett, 1996; Rainey & Larsen, 2002). A number of results also support the view that musical practice improves working memory (Bergman Nutley et al., 2014; Franklin et al., 2008; Hansen et al., 2013; Lee et al., 2007). In WS, verbal memory functioning is atypical. Earlier results suggested an impaired long-term verbal memory while short-term verbal memory would be spared (Volterra et al., 1996). Relatively good phonological memory might explain why individuals with WS are typically fluent and talkative, with some authors suggesting that WS is characterized by a stronger reliance on phonology during language acquisition (Karmiloff-Smith et al., 1997; Majerus et al., 2003; Volterra et al., 1996; but see Brock et al., 2006). Yet, the latter account rather suggests a general developmental delay in verbal memory and that dissociation between short- and long-term systems is not particularly relevant (Brock et al., 2006; Sampaio et al., 2008). The studies by Martens et al. (2011) and Dunning et al. (2015) were dedicated to musical engagement and verbal memory in WS. Their main finding suggests that formal music lessons can improve memory for sung sentences.

Those two studies (Dunning et al., 2015; Martens et al., 2011) are easily comparable as they share very close objectives and methods. Musically trained and untrained WS participants were presented with animal pictures. They first saw pictures of isolated animals, then, of an animal group. They were instructed that a given animal group was called a particular name (e.g., a group of horses is called a band; a group of ducks is called a raft). Most importantly, the instructions were either spoken or sung. Subsequently, they were assessed for their immediate and delayed cued-recall and recognition from a multiple-choice paradigm. Martens et al. (2011) observed better memory for sung sentences than for spoken ones in musically trained WS participants. This beneficial effect of musical lessons was observed for delayed memory (after 15 min) but not for immediate memory. Furthermore, it was observed in the multiple-choice recognition paradigm, but not in the more challenging recall task. The work of Dunning et al. (2015) aimed to determine whether a new melody could also influence verbal memory of individuals with WS. In the study of Martens et al. (2011), sentences were sung to the popular Twinkle, Twinkle Little Star melody. Dunning et al. (2015) created a new, unfamiliar melody based on the same harmonic and structure. They also observed improved memory for musically trained participants, but this time in both learning conditions. Musically trained participants were better than their untrained counterparts at sung and spoken information. The authors stated that new melodies are known to provide significantly better memory improvements in individuals with TD and could have a comparable effect for those with WS (Calvert & Tart, 1993; Crowder et al., 1990; McElhinney & Annett, 1996; Yalch, 1991). Music training had a significant effect only on cued-recall and not on multiple-choice recognition, which is different from the initial work of Martens et al. (2011). Taken together, these studies call for further investigation of musical engagement and its influence on verbal memory. Overall, they suggest that experience with musical practice is beneficial, irrespective of verbal IQ, chronological age, music enjoyment or emotional responsiveness to music.

#### 3.1.4. Musicality and Emotional Processing

Emotional processing in individuals with WS is known to be in line with MA but also come with atypical processing of emotional valence. A specific impairment for negative compared to positive emotion identification is usually observed, both for facial and verbal emotion recognition (Dodd & Porter, 2010; Gagliardi et al., 2003; Lacroix et al., 2009; Plesa-Skwerer et al., 2006; Porter et al., 2007, 2010). Given the strong emotional impact of music, this raises the question of whether the processing of emotions related to speech and music is comparable in individuals with WS. Two studies provide preliminary insights suggesting a relationship between musical artistry (i.e., creativity, expressivity, sensitivity, emotionality) and verbal emotion. Similar to TD, individuals with WS seem to be more accurate when processing vocal than musical emotions. Their decreased sensitivity to negative emotions such as fear and sadness is observed for both vocal and music related emotions. Finally, a particular relationship between emotional responsiveness to music and verbal skills could exist. A strong reverse correlation between emotional responsiveness to music and linguistic competence would characterize individuals with WS, indicating that the greater their linguistic competence, the smaller their emotional responsiveness to music. An opposite pattern is observed in TD.

Heaton et al. (2020) investigated vocal and musical emotion recognition. Their participants had to identify musical excerpts and vocalizations corresponding to happy, sad, fearful and angry stimuli. Importantly, this study was not directly related to verbal but rather paraverbal information. Vocal emotions corresponded to paraverbal vocalizations in line with a given emotion (e.g., crying, laughter). As expected, participants with TD outperformed those with WS and typically, participants with WS were impaired when processing sad and angry emotions. More importantly, both groups were better at identifying vocalized emotions than musical emotions. Moreover, participants with WS exhibited a similar positive bias (i.e., difficulty in processing negative emotion), no matter what the condition. From a broader perspective, processing vocalized and musical emotions seems to be very comparable in WS and TD.

From a different approach, Ng et al. (2013) investigated the correlation between musicality, sociability and verbal skills. Musicality was assessed using the Salk/McGill Music Inventory (Levitin et al., 2004), which has been used in several studies about WS and provides assessment for various subdimensions of musicality. In this work, the authors particularly focused on musical interest, musical creativity, emotional responsiveness to and expressiveness in music. Two main results were reported. Firstly, a positive association was found in participants with WS between emotional responsiveness to music and emotion sensitivity in social interaction. Unlike individuals with TD, the musicality–sociability relationship appears to be a defining feature of individuals with WS. Second, emotional responsiveness to music of participants with WS was inversely correlated with verbal ability measured by the Peabody Picture Vocabulary Test, 3rd edition (Dunn & Dunn, 1997). Musical emotion expressivity was less prevalent in individuals with WS with better linguistic ability, while in TD the opposite pattern was observed, with stronger language capacity for individuals with a stronger musical emotion expressivity. Emotional responsiveness to music and vocal stimuli may therefore be other atypical cognitive features of WS.

### 3.2. Methodological Review

#### 3.2.1. Diagnosis

Diagnosis of WS is currently based on DNA testing using fluorescent in situ hybridization (FISH). FISH has become increasingly available since the 1990s; however, a variety of diagnosis methods based on medical and phenotypical evaluations has been used in the past. In line with current findings in WS research, the works reviewed here exhibited heterogeneity in their diagnostic approaches. Three used a combination of phenotypic and FISH to confirm WS diagnosis: one used a combination of phenotypic and genetic assessment without specifying the method, one used FISH alone, one used either a phenotypic or FISH diagnostic, and two did not indicate any diagnostic method. Even if the diagnostic approaches were not related to methodological quality, a combination of phenotypical and FISH evaluation would be preferred to enhance the scientific rigor of research in WS (Martens et al., 2008; Thakur et al., 2018).

#### 3.2.2. IQ Report

All articles reported measures of verbal IQ. Four of them used the Peabody Picture Vocabulary Test 3rd edition (Dunn & Dunn, 1997), two used the Kaufman Brief Intelligence Test 2nd edition (Kaufman & Kaufman, 2004) and two used the Wechsler Intelligence Scale for Children IV or the Wechsler Intelligence Scale for Adults III, depending on the participant’s age. Four out of eight studies also included nonverbal, composite, full scale or performance IQ measurement based on KBIT-2 or on Wechsler scales. However, the relationship between IQ and musical practice and/or musical skills remains unknown. No articles assessed whether long-term musical practice could increase IQ in children with WS, as is the case in TD (Protzko, 2017; Schellenberg, 2004, 2006; but see also Román-Caballero et al., 2022).

#### 3.2.3. Hearing Loss and Sensitivity

Hearing loss and sensitivities are important features to assess when investigating musical cognition and musicality. Four studies relied on parental reports of hearing sensitivity. Two of them used unspecified parental reports, one used an unspecified parental report to investigate history of otitis and hyperacusis alone, and the last used parents’ answers to the Salk/McGill Music Inventory (Levitin et al., 2004). One of the remaining articles used a pure tone audiometric test to assess normal hearing while the remaining three provide no indication of the methods used. Considering that normal hearing is of crucial importance for music and given the prevalence of specific auditory perception in WS (i.e., hyperacusis, odynacusis, auditory aversion and auditory fascination, (Levitin et al., 2005)), systematic hearing evaluations would be preferable in future studies (Thakur et al., 2018).

#### 3.2.4. Control Group

Most studies compared participants with WS and TD counterparts, with only two out of eight involving solely participants with WS (Table 1). Appropriate matching of participants with TD and WS largely depends on study outcomes and may involve CA or MA as well as other task-relevant abilities such as VMA (Martens et al., 2008). Among the six studies involving TD controls, one involved MA-matched TD, three involved CA-matched TD, one involved CA- and VMA-matched TD and the last one did not specify matching criteria. When investigating the relationship between musical and verbal skills, matching by VMA seems particularly relevant. Studies investigating the beneficial effects of music on language development should, as much as possible, control for verbal mental age (VMA) as a potential confounding factor. Depending on the objectives, other relevant matching criteria may involve musical practice, years of musical experience, emotional responsiveness to music or types of instruments played.

#### 3.2.5. Musicality Assessment

Even though all studies were focused on music, the types of musicality subsets investigated differed greatly. Moreover, depending on the goal of the study, very diverse tools were used to assess musicality. One study used the Salk/McGill Music Inventory (Levitin et al., 2004), and another used the Primary Measures of Music Audiation (E. E. Gordon, 1979) along with a parental interview for musical interest. Four studies used home-made questionnaires to investigate various aspects of musicality including interest in music, music listening, music lessons, music ability and music therapy. Two studies did not report any formal assessment of musicality and only investigated whether music-related experimental conditions benefitted participants with WS. Broadly speaking, studies on WS would be improved by the systematic use of standardized tools to assess musicality. Currently, the Salk/McGill Music Inventory (Levitin et al., 2004) is the only known method to assess a wide range of musicality subdimensions. It is based on 46 items, providing information on demographic background, interest in music, emotional response to music, creativity and reproduction, musical training and age of onset. Unfortunately, the test has not been translated yet and is only available in English, limiting its use in research or clinical purposes. Translation and validation of the Salk/McGill Music Inventory or development of comparable tools would greatly benefit our understanding of musicality in WS.

#### 3.2.6. Statistical Power, Sample Size and Effect Size

WS being a rare disease, researchers regularly struggle to recruit participants. Sample sizes are usually small or medium, sometimes barely sufficient for an appropriate use of parametric tests (Brysbaert, 2019). For example, half of the works included in the present review included less than 20 participants. Small sample sizes are challenging for the validity of experimental results as they have a direct negative influence on statistical power (Button et al., 2013; Cohen, 1992). Statistical power corresponds to the probability that the null hypothesis will be correctly rejected when false, or not committing a type II error. Statistical power depends on sample size, chosen level of significance (α, risk of type I error, which is often set to 5%) and effect size, that is, the discrepancy between the null hypothesis H0 and H1, a standardized measure that quantifies the size of the difference between two groups or the strength of an association between two variables (Button et al., 2013; Cohen, 1992). Generally, a study is regarded as sufficiently powered when false negative probability does not exceed 20% (1 − β = 0.8). Underpowered studies might be an issue in WS research, and several works reviewed here assumed that some of their inconclusive results originated from a lack of statistical power. Unfortunately, as sample size is crucial to improve statistical power and because WS participants are difficult to recruit and test, an ideal solution is not always available. Researchers studying WS arguably tend to overlook sample size estimation, as they are aware that recruitment is typically limited to a relatively small number of participants. Strikingly, none of the studies investigated here conducted a pre-study power analysis. Despite not being fully satisfactory, researchers should consider power analysis, even when resources are limited (Lakens, 2022). Estimating sample size in advance based on an expected effect size remains informative for highlighting the gap between ideal methodological standards and practical constraints. Moreover, retrospective power analysis (i.e., after data gathering) is also an option. Retrospective power analysis sheds light on how much power a study has, given its known sample size and α, and how likely it is to observe an effect if the effect size in the sample corresponds to the effect size in the target population (Aberson, 2011). Retrospective power analysis is of particular interest when reporting null or ambiguous results, as it may help to decide whether they originate from the nonexistence of a hypothesized effect or from an underpowered study (Fagley, 1985; Onwuegbuzie & Leech, 2004). In addition, reporting effect size in experimental articles is regarded as good research practice, as it provides a basis for power analysis in future studies (Lakens, 2013). Of the studies reviewed, six out of eight reported some effect size indicators. Finally, a common strategy to increase statistical power, despite the small number of WS participants, is to recruit larger groups of TD controls. Larger control groups can be an effective way to increase the statistical power of a study in certain contexts (Lydersen, 2018; Oldfield, 2016). However, the control group size should not be increased by more than four- to five-fold, as further increases contribute little to statistical power. (Belle, 2011; Lydersen, 2018). From a methodological perspective, comparing groups of highly unequal sizes may violate parametric assumptions and lead to unacceptable increases in error rates (Riniolo, 1999).

#### 3.2.7. Testing Limitations

- Age ranges of participants with WS were sometimes very large. Four experiments recruited participants from very different age ranges (i.e., 8–48; 9–26; 6–59; 16–52). Whether large age ranges might impact the conclusions of those studies remains a question. Ideally, future studies should stay within group age ranges as closely as possible. This is of particular importance for studies with a stronger focus on developmental issues.
- When comparing musically trained and untrained participants, it would be preferable to determine how many participants to include in both groups before the beginning of the experiment. Some studies recruited participants with WS without prior knowledge on their musical practice, which was incompatible with the previous sample size calculation.
- Considering the prosocial nature of individuals with WS, it was argued that live and recorded musical excerpts could influence them differently (Thakur et al., 2018). They could be more inclined to musical stimuli in social contexts. Such a hypothesis deserves further investigation.
- Systematic assessment of musical engagement (i.e., years of practice) was not always conducted, which may be a potential confound. Studies that are not directly interested in the effect of musical engagement should still assess whether their participants with WS have a history of musical practice, as this could influence their performance in tasks such as pitch discrimination, prosody comprehension or musical emotion responsiveness.
- Several experiments involved discrimination between stimuli that may be categorized as “high vs. low pitch” or “same vs. different”. A full understanding of the concept of ‘pitch’ is not guaranteed in individuals with WS, and some authors have raised concerns that the ‘same vs. different’ distinction may place a burden on their limited verbal memory (Thakur et al., 2018). Pre-testing may be useful to clarify whether participants with WS fully understand the pitch discrimination task. For example, pretests involving various instruments or vocalization may help to ensure a participant reliably discriminates pitch. A comparable pre-testing approach is sometimes used for the “same vs. different” instructions (Hopyan et al., 2001). The task designed by Kitamura et al. (2020) is also of interest, as it was developed specifically to prevent verbal skills hindering pitch discrimination. It relied on correspondence between pitch height and visual motion rather than a verbal label. Lastly, as proposed by Thakur et al. (2018), assessment of verbal memory as a covariate may help to control for a memory load effect.
- As is often emphasized, a correlation exists between musicality and verbal skills in WS, but no conclusion can be drawn yet regarding causal relationships. Evidence showing that musical affinity, experience, engagement, artistry or music perception are directly responsible for changes in verbal abilities is lacking. Children with WS that are prone to adopt musical behavior or with specific affinity for music may improve their verbal skills because of musicality, but it is also possible that a common underlying factor moderates musicality and language in WS. Even if VMA does not influence the relationship between musicality and language in WS, other individual differences might be the cause.

## 4. Discussion

### 4.1. Summary of Main Findings

Although there is still a modest number of studies dedicated to music and language in WS, they provide encouraging results and call for further investigations. So far, evidence suggests moderate correlations between basic musical ability (tone and rhythm discrimination) and several verbal skills including auditory closure and auditory attention (Don et al., 1999). Prosody discrimination for isolated words is correlated with pitch discrimination (Martínez-Castilla & Sotillo, 2014), and musical practice may have a positive effect, particularly because it could increase prosody discrimination for short sentences (Martínez-Castilla et al., 2019). However, it remains unclear whether pitch discrimination develops in parallel with language, as it does in typically developing individuals, or follows an atypical trajectory. (Kitamura et al., 2020; Martínez-Castilla et al., 2016). Music is also linked with verbal memory. Further investigations on musical practice and its influence are necessary, as it seems to benefit WS participants when they memorize sentences (Dunning et al., 2015; Martens et al., 2011). As a final point, musicality provides an opportunity to investigate the specific emotional processing characteristic of WS. Similarly to their TD counterparts, individuals with WS are more accurate when processing vocal (i.e., crying, laughter) than musical emotions. Musical excerpts with a positive emotional valence are easier to identify for them, in line with their known bias for positive over negative emotional stimuli (Heaton et al., 2020). Moreover, emotional responsiveness to music was found to be inversely correlated to verbal ability (Ng et al., 2013).

### 4.2. Research Perspective

The relationship between language and music in WS offers numerous perspectives for new research. The present review highlights several key points that should be kept in mind before investigating the topic. First, the language–music relationship is complex, even in TD. There are reasons to believe that some aspects of language and music involve identical mechanisms (e.g., pitch, tone, rhythm processing), that others involve close and comparable mechanisms (e.g., auditory memory), and that others are quite distinct (e.g., syntaxes) (Brown, 2001; Jackendoff, 2009; Lerdahl & Jackendoff, 1996; Pinker, 1997). Similar mechanisms are of specific interest for future studies as they are more likely to provide results with clinical implications. Second, cognitive functions involved in language and music may sometimes be delayed or develop atypically in WS compared to TD. Consequently, the fact that a cognitive function is involved in music and language in TD does not necessarily mean it is in WS, or possibly not at the same stage of development.

As is frequently emphasized in the literature, it is likely that music benefits only some individuals with WS. It could be particularly useful for those with the mildest prosodic difficulties or the highest musical skills or interests. Comparisons of individuals with WS with good and poor skills would therefore be desirable. In addition, language involves many skills whose relationship to music has never been assessed in WS. For example, in TD, musical practice benefits phonological awareness and literacy acquisition (Degé & Schwarzer, 2011; Flaugnacco et al., 2015; R. L. Gordon et al., 2015). Whether the same is true in WS remains an open question. Finally, among children with TD, musical practice may increase IQ (Protzko, 2017; Schellenberg, 2004, 2006; but see also Román-Caballero et al., 2022). In WS, the music–IQ relationship is largely underexplored. Interestingly, it has been suggested that increased IQ in musically trained children might originate from improved rhythmic skills (Protzko, 2017). Indeed, rhythmic skills are known to correlate with IQ in children with TD (Madison et al., 2009; Rammsayer & Brandler, 2007). Since rhythmic skills in WS seem quite comparable to TD, a deeper investigation of the rhythm–IQ relationship in musically trained and untrained children with WS would be of interest. Such an investigation could be valuable for future therapeutic interventions. However, similar to what is needed in individuals with TD, extensive longitudinal studies are also required to understand whether music lessons truly lead to cognitive improvements, or whether they reflect pre-existing differences (Román-Caballero et al., 2022).

It is important to acknowledge, in closing, that the present paper comes with its own limitations. We chose to combine the systematic and integrative review methods. This approach seemed necessary because of the relatively small number of papers dedicated to the topic. It also proved useful in a previous study on WS (Thakur et al., 2018). However, systematic and integrative reviews involve very different methods and are not easy to combine. Due to our integrative approach, we compared papers with varying methods and objectives. Some were dedicated to core aspects of language such as prosody, while others explored the influence of music on more transversal cognitive abilities like verbal memory or verbal emotion processing. Ideally, a fully integrative review would be conducted on both in the future. We sought to mirror the guidelines of systematic reviews to enhance the overall quality and decrease the risk of bias, but given the limited number of articles, some recommendations were hard to follow. For example, exploring the possible cause for heterogeneity among results is challenging in such situations.

## 5. Conclusions

This mini review highlights the relationship between language and music in WS. The results suggested that music might be helpful to improve language skills, especially in relation to prosody understanding or verbal memory. The music–language relationship remains underexplored in WS but is likely to provide meaningful results for a variety of domains.

## Figures and Tables

**Table 1:** Included articles and their topics of interest.

Topic	Authors/Year	*N*	Age (in Years and Months)	Main Finding(s)
Tonal, rhythmic skills and overall language ability	(Don et al., 1999)	WS = 18 TD = 19	WS = 10.6 TD (MA*) = 7.11	WS better in tonal and rhythm tasks than prediction based on full scale, verbal or performance IQ Correlation between musical and verbal performance
Pitch discrimination and prosodic skills	(Kitamura et al., 2020)	VS = 11 TD = 138	WS = 13 TD (unspecified) = 6	Atypical development of pitch discrimination Interest of nonverbal task for pitch discrimination
Pitch discrimination and prosodic skills	(Martínez-Castilla & Sotillo, 2014)	WS = 14 TD = 26	WS = 13.6 TD (CA^α^) = 13.6	Correlation between discrimination of pitch and prosody for isolated words
Pitch discrimination and prosodic skills	(Martínez-Castilla et al., 2019)	WS = 21 TD = 42	WS = 20.1 TD (CA^α^) = 20.2	Musically trained WS outperform untrained counterparts in prosody discrimination
Musicality and verbal Memory	(Martens et al., 2011)	WS = 38	WS = 20.42	WS participants with history of formal musical lesson outperform untrained counterparts in memory task about sung sentences
Musicality and verbal Memory	(Dunning et al., 2015)	WS = 44	WS = 22.11	Improved cued recall of sung and spoken sentences for WS participants with history of formal musical lesson Interest of novel melody
Musicality and emotional processing	(Heaton et al., 2020)	WS = 15 TD (CA) = 18 TD (VMA) = 19	WS = 12 TD (CA^α^) = 11 TD (VMA^β^) = 6	Better identification of vocalized than musical emotion Better identification of positive than negative musical emotion
Musicality and emotional processing	(Ng et al., 2013)	WS = 55 TD = 19	WS = 29.5 TD (CA^α^) = 28.2	Reverse correlation between emotional responsiveness to music and verbal ability

WS = Williams syndrome, TD = typically developed. Abbreviation following TD indicates matching method, MA* = Mental age, CA^α^ = Chronological age, VMA^β^ = Verbal mental age.

## Data Availability

The data presented in this study are openly available at https://osf.io/prc98/ accessed on 24 April 2025.

## Supplementary Materials

The supporting information can be downloaded at https://osf.io/prc98/, accessed on 24 April 2025.

## Abbreviations

The following abbreviations are used in this manuscript:

WS	Williams syndrome
TD	Typically developed
MA	Mental age
CA	Chronological age
VMA	Verbal mental age
PEPS-C	Prosody in speech communication battery
FISH	Fluorescent in situ hybridization

## References

[B1-behavsci-15-00595] Aberson C. L. (2011). Applied power analysis for the behavioral sciences.

[B2-behavsci-15-00595] Arbib M. A. (2013). Language, music, and the brain: A mysterious relationship.

[B3-behavsci-15-00595] Belle G. (2011). Statistical rules of thumb.

[B4-behavsci-15-00595] Bello A., Capirci O., Volterra V. (2004). Lexical production in children with Williams syndrome: Spontaneous use of gesture in a naming task. Neuropsychologia.

[B5-behavsci-15-00595] Berdon W. E., Clarkson P. M., Teele R. L. (2011). Williams-Beuren syndrome: Historical aspects. Pediatric Radiology.

[B6-behavsci-15-00595] Bergman Nutley S., Darki F., Klingberg T. (2014). Music practice is associated with development of working memory during childhood and adolescence. Frontiers in Human Neuroscience.

[B7-behavsci-15-00595] Berz W. L. (1995). Working memory in music: A theoretical model. Music Perception.

[B8-behavsci-15-00595] Besson M., Chobert J., Marie C. (2011). Transfer of training between music and speech: Common processing, attention, and memory. Frontiers in Psychology.

[B9-behavsci-15-00595] Besson M., Schön D. (2001). Comparison between language and music. Annals of the New York Academy of Sciences.

[B10-behavsci-15-00595] Beuren A. J., Apitz J., Harmjanz D. (1962). Supravalvular aortic stenosis in association with mental retardation and a certain facial appearance. Circulation.

[B11-behavsci-15-00595] Brock J., Brown G. D. A., Boucher J. (2006). Free recall in Williams syndrome: Is there a dissociation between short-and long-term memory?. Cortex; a Journal Devoted to the Study of the Nervous System and Behavior.

[B12-behavsci-15-00595] Brown S. (2001). Are music and language homologues?. Annals of the New York Academy of Sciences.

[B13-behavsci-15-00595] Brysbaert M. (2019). How many participants do we have to include in properly powered experiments? A tutorial of power analysis with reference tables. Journal of Cognition.

[B14-behavsci-15-00595] Button K. S., Ioannidis J. P. A., Mokrysz C., Nosek B. A., Flint J., Robinson E. S. J., Munafò M. R. (2013). Power failure: Why small sample size undermines the reliability of neuroscience. Nature Reviews Neuroscience.

[B15-behavsci-15-00595] Calvert S. L., Tart M. (1993). Song versus verbal forms for very-long-term, long-term, and short-term verbatim recall. Journal of Applied Developmental Psychology.

[B16-behavsci-15-00595] Cohen J. (1992). Statistical power analysis. Current Directions in Psychological Science.

[B17-behavsci-15-00595] Crowder R. G., Serafine M. L., Repp B. (1990). Physical interaction and association by contiguity in memory for the words and melodies of songs. Memory & Cognition.

[B18-behavsci-15-00595] Degé F., Schwarzer G. (2011). The effect of a music program on phonological awareness in preschoolers. Frontiers in Psychology.

[B19-behavsci-15-00595] Deutsch D., Henthorn T., Dolson M. (2004). Absolute pitch, speech, and tone language: Some experiments and a proposed framework. Music Perception.

[B20-behavsci-15-00595] Dodd H. F., Porter M. A. (2010). I see happy people: Attention bias towards happy but not angry facial expressions in Williams syndrome. Cognitive Neuropsychiatry.

[B21-behavsci-15-00595] Don A. J., Schellenberg G. E., Rourke B. P. (1999). Music and language skills of children with williams syndrome. Child Neuropsychology.

[B22-behavsci-15-00595] Dunn L. M., Dunn L. M. (1997). Peabody picture vocabulary test. 3.

[B23-behavsci-15-00595] Dunning B. A., Martens M. A., Jungers M. K. (2015). Music lessons are associated with increased verbal memory in individuals with Williams syndrome. Research in Developmental Disabilities.

[B24-behavsci-15-00595] Ewart A. K., Morris C. A., Atkinson D., Jin W., Sternes K., Spallone P., Stock A. D., Leppert M., Keating M. T. (1993). Hemizygosity at the elastin locus in a developmental disorder, Williams syndrome. Nature Genetics.

[B25-behavsci-15-00595] Fadiga L., Craighero L., D’Ausilio A. (2009). Broca’s area in language, action, and music. Annals of the New York Academy of Sciences.

[B26-behavsci-15-00595] Fagley N. S. (1985). Applied statistical power analysis and the interpretation of nonsignificant results by research consumers. Journal of Counseling Psychology.

[B27-behavsci-15-00595] Farran E. K., Jarrold C. (2003). Visuospatial cognition in Williams syndrome: Reviewing and accounting for the strengths and weaknesses in performance. Developmental Neuropsychology.

[B28-behavsci-15-00595] Fennell A. M., Bugos J. A., Payne B. R., Schotter E. R. (2021). Music is similar to language in terms of working memory interference. Psychonomic Bulletin & Review.

[B29-behavsci-15-00595] Fiveash A., Bedoin N., Gordon R. L., Tillmann B. (2021). Processing rhythm in speech and music: Shared mechanisms and implications for developmental speech and language disorders. Neuropsychology.

[B30-behavsci-15-00595] Flaugnacco E., Lopez L., Terribili C., Montico M., Zoia S., Schön D. (2015). Music training increases phonological awareness and reading skills in developmental Dyslexia: A randomized control trial. PLOS ONE.

[B31-behavsci-15-00595] Franklin M. S., Moore K. S., Yip C.-Y., Jonides J., Rattray K., Moher J. (2008). The effects of musical training on verbal memory. Psychology of Music.

[B32-behavsci-15-00595] Gagliardi C., Frigerio E., Burt D. M., Cazzaniga I., Perrett D. I., Borgatti R. (2003). Facial expression recognition in Williams syndrome. Neuropsychologia.

[B33-behavsci-15-00595] Gold C., Voracek M., Wigram T. (2004). Effects of music therapy for children and adolescents with psychopathology: A meta-analysis. Journal of Child Psychology and Psychiatry.

[B34-behavsci-15-00595] Gordon E. E. (1979). Developmental music aptitude as measured by the primary measures of music audiation. Psychology of Music.

[B35-behavsci-15-00595] Gordon R. L., Fehd H. M., McCandliss B. D. (2015). Does music training enhance literacy skills? A meta-analysis. Frontiers in Psychology.

[B36-behavsci-15-00595] Hallam S., Cross I., Thaut M. (2011). Oxford handbook of music psychology.

[B37-behavsci-15-00595] Hansen M., Wallentin M., Vuust P. (2013). Working memory and musical competence of musicians and non-musicians. Psychology of Music.

[B38-behavsci-15-00595] Heaton P., Ridley E., Makhmood S., Riby D. M. (2020). Hearing the feeling: Auditory emotion perception in Williams syndrome. Research in Developmental Disabilities.

[B39-behavsci-15-00595] Holinger D. P., Bellugi U., Mills D. L., Korenberg J. R., Reiss A. L., Sherman G. F., Galaburda A. M. (2005). Relative sparing of primary auditory cortex in Williams Syndrome. Brain Research.

[B40-behavsci-15-00595] Hopyan T., Dennis M., Weksberg R., Cytrynbaum C. (2001). Music skills and the expressive interpretation of music in children with Williams-Beuren syndrome: Pitch, rhythm, melodic imagery, phrasing, and musical affect. Child Neuropsychology.

[B41-behavsci-15-00595] Ito K., Martens M. A. (2017). Contrast-marking prosodic emphasis in Williams syndrome: Results of detailed phonetic analysis. International Journal of Language & Communication Disorders.

[B42-behavsci-15-00595] Jackendoff R. (2009). Parallels and nonparallels between language and music. Music Perception: An Interdisciplinary Journal.

[B43-behavsci-15-00595] Jarrold C., Baddeley A. D., Hewes A. K. (1999). Genetically dissociated components of working memory: Evidence from Downs and Williams syndrome. Neuropsychologia.

[B44-behavsci-15-00595] Järvinen-Pasley A., Vines B. W., Hill K. J., Yam A., Grichanik M., Mills D., Reiss A. L., Korenberg J. R., Bellugi U. (2010). Cross-modal influences of affect across social and non-social domains in individuals with Williams syndrome. Neuropsychologia.

[B45-behavsci-15-00595] Karmiloff-Smith A., Grant J., Berthoud I., Davies M., Howlin P., Udwin O. (1997). Language and Williams syndrome: How intact is “Intact”?. Child Development.

[B46-behavsci-15-00595] Kasdan A., Gordon R. L., Lense M. D. (2022). Neurophysiological correlates of dynamic beat tracking in individuals with Williams syndrome. Biological Psychiatry: Cognitive Neuroscience and Neuroimaging.

[B47-behavsci-15-00595] Kaufman A. S., Kaufman N. L. (2004). Kaufman brief intelligence test.

[B48-behavsci-15-00595] Kitamura Y., Kita Y., Okumura Y., Kaga Y., Okuzumi H., Ishikawa Y., Nakamura M., Inagaki M. (2020). Discrepancy between musical ability and language skills in children with Williams syndrome. Brain & Development.

[B49-behavsci-15-00595] Klein B. P., Mervis C. B. (1999). Contrasting patterns of cognitive abilities of 9-and 10-year-olds with Williams syndrome or down syndrome. Developmental Neuropsychology.

[B50-behavsci-15-00595] Koelsch S., Kasper E., Sammler D., Schulze K., Gunter T., Friederici A. D. (2004). Music, language and meaning: Brain signatures of semantic processing. Nature Neuroscience.

[B51-behavsci-15-00595] Kraus N., Slater J., Aminoff M. J., Boller F., Swaab D. F. (2015). Chapter 12—Music and language: Relations and disconnections. Handbook of clinical neurology.

[B52-behavsci-15-00595] Lacroix A., Guidetti M., Rogé B., Reilly J. (2009). Recognition of emotional and nonemotional facial expressions: A comparison between Williams syndrome and autism. Research in Developmental Disabilities.

[B53-behavsci-15-00595] Lakens D. (2013). Calculating and reporting effect sizes to facilitate cumulative science: A practical primer for *t*-tests and ANOVAs. Frontiers in Psychology.

[B54-behavsci-15-00595] Lakens D. (2022). Sample size justification. Collabra: Psychology.

[B55-behavsci-15-00595] Lee Y., Lu M., Ko H. (2007). Effects of skill training on working memory capacity. Learning and Instruction.

[B56-behavsci-15-00595] Lense M. D., Dykens E. M. (2016). Beat perception and sociability: Evidence from Williams syndrome. Frontiers in Psychology.

[B57-behavsci-15-00595] Lense M. D., Ladányi E., Rabinowitch T.-C., Trainor L., Gordon R. (2021). Rhythm and timing as vulnerabilities in neurodevelopmental disorders. Philosophical Transactions of the Royal Society B: Biological Sciences.

[B58-behavsci-15-00595] Lerdahl F., Jackendoff R. S. (1996). A generative theory of tonal music.

[B59-behavsci-15-00595] Levitin D. J., Cole K., Chiles M., Lai Z., Lincoln A., Bellugi U. (2004). Characterizing the musical phenotype in individuals with Williams Syndrome. Child Neuropsychology.

[B60-behavsci-15-00595] Levitin D. J., Cole K., Lincoln A., Bellugi U. (2005). Aversion, awareness, and attraction: Investigating claims of hyperacusis in the Williams syndrome phenotype. Journal of Child Psychology and Psychiatry.

[B61-behavsci-15-00595] Linnavalli T., Putkinen V., Lipsanen J., Huotilainen M., Tervaniemi M. (2018). Music playschool enhances children’s linguistic skills. Scientific Reports.

[B62-behavsci-15-00595] Loveall S. J., Hawthorne K., Gaines M. (2021). A meta-analysis of prosody in autism, Williams syndrome, and Down syndrome. Journal of Communication Disorders.

[B63-behavsci-15-00595] Lydersen S. (2018). Balanced or imbalanced samples?.

[B64-behavsci-15-00595] Madison G., Forsman L., Blom Ö., Karabanov A., Ullén F. (2009). Correlations between intelligence and components of serial timing variability. Intelligence.

[B65-behavsci-15-00595] Magne C., Schön D., Besson M. (2006). Musician Children Detect Pitch Violations in Both Music and Language Better than Nonmusician Children: Behavioral and Electrophysiological Approaches. Journal of Cognitive Neuroscience.

[B66-behavsci-15-00595] Majerus S., Barisnikov K., Vuillemin I., Poncelet M., Linden M. (2003). An investigation of verbal short-term memory and phonological processing in four children with Williams syndrome. Neurocase.

[B67-behavsci-15-00595] Mampe B., Friederici A. D., Christophe A., Wermke K. (2009). Newborns’ cry melody is shaped by their native language. Current Biology.

[B68-behavsci-15-00595] Martens M. A., Jungers M. K., Steele A. L. (2011). Effect of musical experience on verbal memory in Williams syndrome: Evidence from a novel word learning task. Neuropsychologia.

[B69-behavsci-15-00595] Martens M. A., Wilson S. J., Reutens D. C. (2008). Research review: Williams syndrome: A critical review of the cognitive, behavioral, and neuroanatomical phenotype. Journal of Child Psychology and Psychiatry and Allied Disciplines.

[B70-behavsci-15-00595] Martínez-Castilla P., Campos R., Sotillo M. (2019). Enhanced linguistic prosodic skills in musically trained individuals with Williams syndrome. Language and Cognition.

[B71-behavsci-15-00595] Martínez-Castilla P., Peppé S. (2008). Developing a test of prosodic ability for speakers of Iberian Spanish. Speech Communication.

[B72-behavsci-15-00595] Martínez-Castilla P., Rodríguez M., Campos R. (2016). Developmental trajectories of pitch-related music skills in children with Williams syndrome. Research in Developmental Disabilities.

[B73-behavsci-15-00595] Martínez-Castilla P., Sotillo M. (2014). Pitch processing in children with Williams syndrome: Relationships between music and prosody skills. Brain Sciences.

[B74-behavsci-15-00595] Mastnak W., Neuwirthová A. (2017). Children with Williams syndrome make music: A community-based care model in the Czech Republic. International Journal of Community Music.

[B75-behavsci-15-00595] McElhinney M., Annett J. M. (1996). Pattern of efficacy of a musical mnemonic on recall of familiar words over several presentations. Perceptual and Motor Skills.

[B76-behavsci-15-00595] Mcmullen E., Saffran J. R. (2004). Music and language: A developmental comparison. Music Perception.

[B77-behavsci-15-00595] Mervis C. B., Robinson B. F., Bertrand J., Morris C. A., Klein-Tasman B. P., Armstrong S. C. (2000). The Williams syndrome cognitive profile. Brain and Cognition.

[B78-behavsci-15-00595] Miezah D., Porter M., Batchelor J., Boulton K., Campos Veloso G. (2020). Cognitive abilities in Williams syndrome. Research in Developmental Disabilities.

[B79-behavsci-15-00595] Moher D., Liberati A., Tetzlaff J., Altman D. G. (2010). Preferred reporting items for systematic reviews and meta-analyses: The PRISMA statement. International Journal of Surgery.

[B80-behavsci-15-00595] Moher D., Shamseer L., Clarke M., Ghersi D., Liberati A., Petticrew M., Shekelle P., Stewart L. A., PRISMA-P Group (2015). Preferred reporting items for systematic review and meta-analysis protocols (PRISMA-P) 2015 statement. Systematic Reviews.

[B81-behavsci-15-00595] Moore B. C. J. (1995). Hearing.

[B82-behavsci-15-00595] Morris C. A., Demsey S. A., Leonard C. O., Dilts C., Blackburn B. L. (1988). Natural history of Williams syndrome: Physical characteristics. The Journal of Pediatrics.

[B83-behavsci-15-00595] Nazzi T., Gopnik A., Karmiloff-Smith A. (2005). Asynchrony in the cognitive and lexical development of young children with Williams syndrome. Journal of Child Language.

[B84-behavsci-15-00595] Nazzi T., Paterson S., Karmiloff-Smith A. (2003). Early word segmentation by infants and toddlers with Williams syndrome. Infancy.

[B85-behavsci-15-00595] Neves L., Correia A. I., Castro S. L., Martins D., Lima C. F. (2022). Does music training enhance auditory and linguistic processing? A systematic review and meta-analysis of behavioral and brain evidence. Neuroscience & Biobehavioral Reviews.

[B86-behavsci-15-00595] Ng R., Lai P., Levitin D. J., Bellugi U. (2013). Musicality correlates with sociability and emotionality in Williams syndrome. Journal of Mental Health Research in Intellectual Disabilities.

[B87-behavsci-15-00595] Oldfield M. (2016). Unequal sample sizes and the use of larger control groups pertaining to power of a study. Dstl.

[B88-behavsci-15-00595] Ong A., Namasivayam-MacDonald A., Kim S., Werden Abrams S. (2024). The use of music and music-related elements in speech-language therapy interventions for adults with neurogenic communication impairments: A scoping review. International Journal of Language & Communication Disorders.

[B89-behavsci-15-00595] Onwuegbuzie A. J., Leech N. L. (2004). Post hoc power: A concept whose time has come. Understanding Statistics.

[B90-behavsci-15-00595] Oxenham A. J. (2012). Pitch perception. Journal of Neuroscience.

[B91-behavsci-15-00595] Patel A. D. (2003). Language, music, syntax and the brain. Nature Neuroscience.

[B92-behavsci-15-00595] Pfordresher P. Q., Brown S. (2009). Enhanced production and perception of musical pitch in tone language speakers. Attention, Perception, & Psychophysics.

[B93-behavsci-15-00595] Pinker S. (1997). How the mind works *(pp. xii, 660)*.

[B94-behavsci-15-00595] Plesa-Skwerer D., Faja S., Schofield C., Verbalis A., Tager-Flusberg H. (2006). Perceiving facial and vocal expressions of emotion in individuals With Williams syndrome. American Journal on Mental Retardation.

[B95-behavsci-15-00595] Porter M. A., Coltheart M., Langdon R. (2007). The neuropsychological basis of hypersociability in Williams and Down syndrome. Neuropsychologia.

[B96-behavsci-15-00595] Porter M. A., Shaw T. A., Marsh P. J. (2010). An unusual attraction to the eyes in Williams-Beuren syndrome: A manipulation of facial affect while measuring face scanpaths. Cognitive Neuropsychiatry.

[B97-behavsci-15-00595] Protzko J. (2017). Raising IQ among school-aged children: Five meta-analyses and a review of randomized controlled trials. Developmental Review.

[B98-behavsci-15-00595] Rainey D. W., Larsen J. D. (2002). The effect of familiar melodies on initial learning and long-term memory for unconnected text. Music Perception.

[B99-behavsci-15-00595] Rammsayer T. H., Brandler S. (2007). Performance on temporal information processing as an index of general intelligence. Intelligence.

[B100-behavsci-15-00595] Reis S. M., Schader R., Milne H., Stephens R. (2003). Music & minds: Using a talent development approach for young adults with Williams syndrome. Exceptional Children.

[B101-behavsci-15-00595] Riniolo T. C. (1999). Using a large control group for statistical comparison: Evaluation of a between-groups median test. The Journal of Experimental Education.

[B102-behavsci-15-00595] Román-Caballero R., Vadillo M. A., Trainor L. J., Lupiáñez J. (2022). Please don’t stop the music: A meta-analysis of the cognitive and academic benefits of instrumental musical training in childhood and adolescence. Educational Research Review.

[B103-behavsci-15-00595] Romero-Rivas C., Rodríguez-Cuadrado S., Sabater L., Gómez P. R., de la Guía I. H., Moreno E. M., Heinze E. G. (2023). Beyond the conservative hypothesis: A meta-analysis of lexical-semantic processing in Williams syndrome. Language and Cognition.

[B104-behavsci-15-00595] Sadiqzade Z. (2024). The impact of music on language learning: A harmonious path to mastery. EuroGlobal Journal of Linguistics and Language Education.

[B105-behavsci-15-00595] Sampaio A., Sousa N., Férnandez M., Vasconcelos C., Shenton M. E., Gonçalves Ó. F. (2008). MRI assessment of superior temporal gyrus in Williams syndrome. Cognitive and Behavioral Neurology.

[B106-behavsci-15-00595] Schellenberg E. G. (2004). Music lessons enhance IQ. Psychological Science.

[B107-behavsci-15-00595] Schellenberg E. G. (2006). Long-term positive associations between music lessons and IQ. Journal of Educational Psychology.

[B108-behavsci-15-00595] Schellenberg E. G., Weiss M. W. (2013). Music and cognitive abilities. The psychology of music.

[B109-behavsci-15-00595] Shi Z.-M., Lin G.-H., Xie Q. (2016). Effects of music therapy on mood, language, behavior, and social skills in children with autism: A meta-analysis. Chinese Nursing Research.

[B110-behavsci-15-00595] Siegmüller J. (2004). Williams syndrome across languages.

[B111-behavsci-15-00595] Singer Harris N. G., Bellugi U., Bates E., Jones W., Rossen M. (1997). Contrasting profiles of language development in children with williams and down syndromes. Developmental Neuropsychology.

[B112-behavsci-15-00595] Slevc L. R. (2012). Language and music: Sound, structure, and meaning. WIREs Cognitive Science.

[B113-behavsci-15-00595] Stojanovik V. (2010). Understanding and production of prosody in children with Williams syndrome: A developmental trajectory approach. Journal of Neurolinguistics.

[B114-behavsci-15-00595] Strømme P., Bjørnstad P. G., Ramstad K. (2002). Prevalence estimation of Williams syndrome. Journal of Child Neurology.

[B115-behavsci-15-00595] Swaminathan S., Schellenberg E. G. (2020). Musical ability, music training, and language ability in childhood. Journal of Experimental Psychology: Learning, Memory, and Cognition.

[B116-behavsci-15-00595] Thakur D., Martens M. A., Smith D. S., Roth E. (2018). Williams syndrome and music: A systematic integrative review. Frontiers in Psychology.

[B117-behavsci-15-00595] Thomas M. S. C., Dockrell J. E., Messer D., Parmigiani C., Ansari D., Karmiloff-Smith A. (2006). Speeded naming, frequency and the development of the lexicon in Williams syndrome. Language and Cognitive Processes.

[B118-behavsci-15-00595] Vicari S., Bates E., Caselli M. C., Pasqualetti P., Gagliardi C., Tonucci F., Volterra V. (2004). Neuropsychological profile of Italians with Williams syndrome: An example of a dissociation between language and cognition?. Journal of the International Neuropsychological Society.

[B119-behavsci-15-00595] Volterra V., Capirci O., Pezzini G., Sabbadini L., Vicari S. (1996). Linguistic abilities in italian children with Williams syndrome. Cortex.

[B120-behavsci-15-00595] Wang P. P., Bellugi U. (1994). Evidence from two genetic syndromes for a dissociation between verbal and visual-spatial short-term memory. Journal of Clinical and Experimental Neuropsychology.

[B121-behavsci-15-00595] Whipple J. (2004). Music in intervention for children and adolescents with autism: A meta-analysis. Journal of Music Therapy.

[B122-behavsci-15-00595] Williams J. C. P., Barratt-Boyes B. G., Lowe J. B. (1961). Supravalvular aortic stenosis. Circulation.

[B123-behavsci-15-00595] Williamson V. J., Baddeley A. D., Hitch G. J. (2010). Musicians’ and nonmusicians’ short-term memory for verbal and musical sequences: Comparing phonological similarity and pitch proximity. Memory & Cognition.

[B124-behavsci-15-00595] Yalch R. F. (1991). Memory in a jingle jungle: Music as a mnemonic device in communicating advertising slogans. Journal of Applied Psychology.

[B125-behavsci-15-00595] Zheng Y., Samuel A. G. (2018). The effects of ethnicity, musicianship, and tone language experience on pitch perception. Quarterly Journal of Experimental Psychology (2006).

